# STING-mediated tumor microenvironment remodeling: turning adaptive resistance into therapeutic vulnerability

**DOI:** 10.3389/fimmu.2026.1937129

**Published:** 2026-09-21

**Authors:** Akash Boda, Elisabetta Bolli, Ahmad Salameh, Christine Gagliardi, Michael A. Curran, Manuela Iezzi, Laura Conti, Federica Cavallo, Federica Pericle

**Affiliations:** 1 ImmunoGenesis, Inc., Houston, TX, United States; 2 Laboratory of OncoImmunology, Molecular Biotechnology Center “Guido Tarone”, Department of Molecular Biotechnology and Health Sciences, University of Turin, Turin, Italy; 3 Department of Immunology, The University of Texas MD Anderson Cancer Center, Houston, TX, United States; 4 Laboratory of Experimental Pathology and Precision Medicine, Center for Advanced Studies and Technology (CAST), Department of Neurosciences, Imaging and Clinical Sciences, “G. d’Annunzio” University of Chieti-Pescara, Chieti, Italy; 5 Eusoma Breast Centre, Department of Pathology, “G. Bernabeo” Hospital Ortona, ASL2 Abruzzo, Ortona, Italy

**Keywords:** Fc engineering, PD-L1/PD-L2, precision immuno-oncology, STING agonist, tumor microenvironment

## Abstract

Resistance to cancer immunotherapy is frequently sustained by immune exclusion, suppressive myeloid and stromal programs, abnormal tumor vasculature, and inhibitory checkpoint-ligand expression within the tumor microenvironment. Activation of the cGAS-STING (cyclic GMP-AMP Synthase)- (Stimulator of Interferon Genes) pathway has emerged as a context-dependent strategy to modulate the cancer immunity cycle and remodel resistant tumor ecosystems, with the potential to convert selected poorly inflamed immune-excluded tumors into immune-reactive lesions. However, STING activation does not simply “turn on” antitumor immunity. By reprogramming the tumor microenvironment, it can generate both therapeutic opportunity and compensatory resistance, including the induction of PD-L1 and PD-L2 on tumor, immune cells, stroma, and endothelial compartments. Recent evidence suggests that this adaptive response may be therapeutically exploited rather than merely counteracted. STING-driven remodeling may sensitize non-malignant tumor microenvironment compartments, including endothelial and myeloid cells, to Fc-engineered antibodies targeting PD-L1 and PD-L2. This Perspective discusses STING agonism as a broad immuno-oncology priming strategy and reframes inducible PD-L1/PD-L2 expression from an adaptive escape mechanism to an emergent transient, spatially organized, and therapeutically actionable cancer selective target. We propose that rational combinations of STING agonists with Fc-engineered PD-L1/PD-L2-targeting antibodies, guided by dynamic and spatial biomarkers, could overcome resistance to conventional immune checkpoint blockade in tumors with minimal T-cell infiltration but targetable tumor vasculature. Emerging delivery platforms, including antibody-conjugated STING agonists, may further improve the therapeutic index of this approach by concentrating innate immune activation within antigen-defined tumor niches and reducing off-tumor inflammatory activation.

## 1 Introduction

Immune checkpoint inhibitors have transformed cancer therapy, yet many tumors remain intrinsically unresponsive or eventually acquire resistance, reflecting diverse mechanisms of immune evasion and tumor evolution. Increasing evidence indicates that this resistance cannot be understood solely through tumor-cell-intrinsic features, such as mutational burden, antigenicity, or oncogenic signaling, but must also be interpreted within the broader tumor microenvironment (TME). This Perspective proposes that Stimulator of Interferon Genes (STING) activation should be viewed as a context-dependent mechanism of TME reconfiguration. Rather than simply amplifying innate antitumor immunity, STING agonism may transiently reshape immune, stromal, myeloid, and endothelial compartments, generating adaptive resistance while also creating novel, therapeutically actionable vulnerabilities. We discuss how STING-induced programmed death ligand 1 (PD-L1) and programmed death ligand 2 (PD-L2) expression may be reframed from a compensatory immune escape mechanism into an emergent cancer-selective targetable state when paired with Fc-engineered checkpoint-ligand antibodies.

### 1.1 Immunotherapy resistance as an ecosystem-level problem

The binary classification of tumors as “hot” or “cold” has provided a useful shorthand, but it obscures the complexity of immune resistance. Tumors that respond to immune checkpoint blockade (ICB) often display a T cell-inflamed phenotype, with lymphocytic infiltration extending into the tumor parenchyma. By contrast, non-responsive tumors may include immune-excluded lesions — in which T cells accumulate peripherally but cannot penetrate the malignant tissue — and immune-desert tumors, in which effective T-cell priming or recruitment is limited (1). Immune exclusion is a frequent pattern across several common tumor types, including colorectal, ovarian, and non-small cell lung cancers. Moreover, although T-cell infiltration was initially considered a reliable surrogate for responsiveness to ICB, it is now clear that even highly infiltrated tumors may resist treatment when the surrounding TME is sufficiently suppressive (2, 3).

This distinction is therapeutically important because it shifts the central question from the extent of T-cell infiltration to the composition and functional state of the TME. Immune checkpoint antibodies can reinvigorate dysfunctional T-cell responses and restore antitumor effector functions, but their activity depends on the presence of a permissive immune contexture. In immune-excluded and immune-desert tumors, inhibitory checkpoint signaling is only one component of resistance. T cells may fail to reach the malignant cells, may be inadequately primed, or may enter a tumor bed dominated by stromal, vascular, and myeloid barriers that prevent productive antitumor immunity (4).

The concerted actions of suppressive tumor-associated macrophages (TAMs) and myeloid-derived suppressor cells (MDSCs) contribute to this resistance by producing anti-inflammatory cytokines, imposing metabolic restrictions, and limiting effector immune-cell access and function (5). However, the vascular compartment is equally central to immune exclusion. Tumor endothelial cells form the anatomical interface through which circulating lymphocytes must pass to enter malignant tissue, but in many tumors this interface is immunologically non-permissive (6, 7). Aberrant angiogenic signaling, particularly through VEGF and related pathways, promotes structurally abnormal vessels, hypoperfusion, hypoxia, and elevated interstitial pressure, all of which can limit immune-cell infiltration (8). In addition, angiogenic factors can induce endothelial anergy, a state in which tumor endothelial cells fail to adequately respond to inflammatory cues and do not efficiently upregulate adhesion molecules such as ICAM-1, VCAM-1, and selectins (9). As a result, even when tumor-reactive T cells are primed and present in the circulation, their adhesion to the vascular wall, extravasation, and penetration into the tumor parenchyma may remain inefficient (6, 10). Although anti-angiogenic and vascular-normalizing therapies can partially overcome this barrier and have provided an important rationale for combinations with immunotherapy (7, 8), the endothelial compartment remains an underexploited target in precision immuno-oncology. Most current immunotherapeutic strategies primarily aim to reinvigorate T cells or deplete/reprogram suppressive immune cells, whereas fewer approaches directly address the tumor endothelial programs that restrict T-cell entry (7). Pathologically activated cancer-associated fibroblasts (CAFs) can further reinforce this barrier by generating dense stromal networks that act as physical and biochemical obstacles to immune infiltration (11). Together, myeloid, endothelial, and stromal components form an ecosystem-level restriction of antitumor immunity (**Figure 1A**) that may require ecosystem-level pharmacologic interventions.

**Figure 1:**
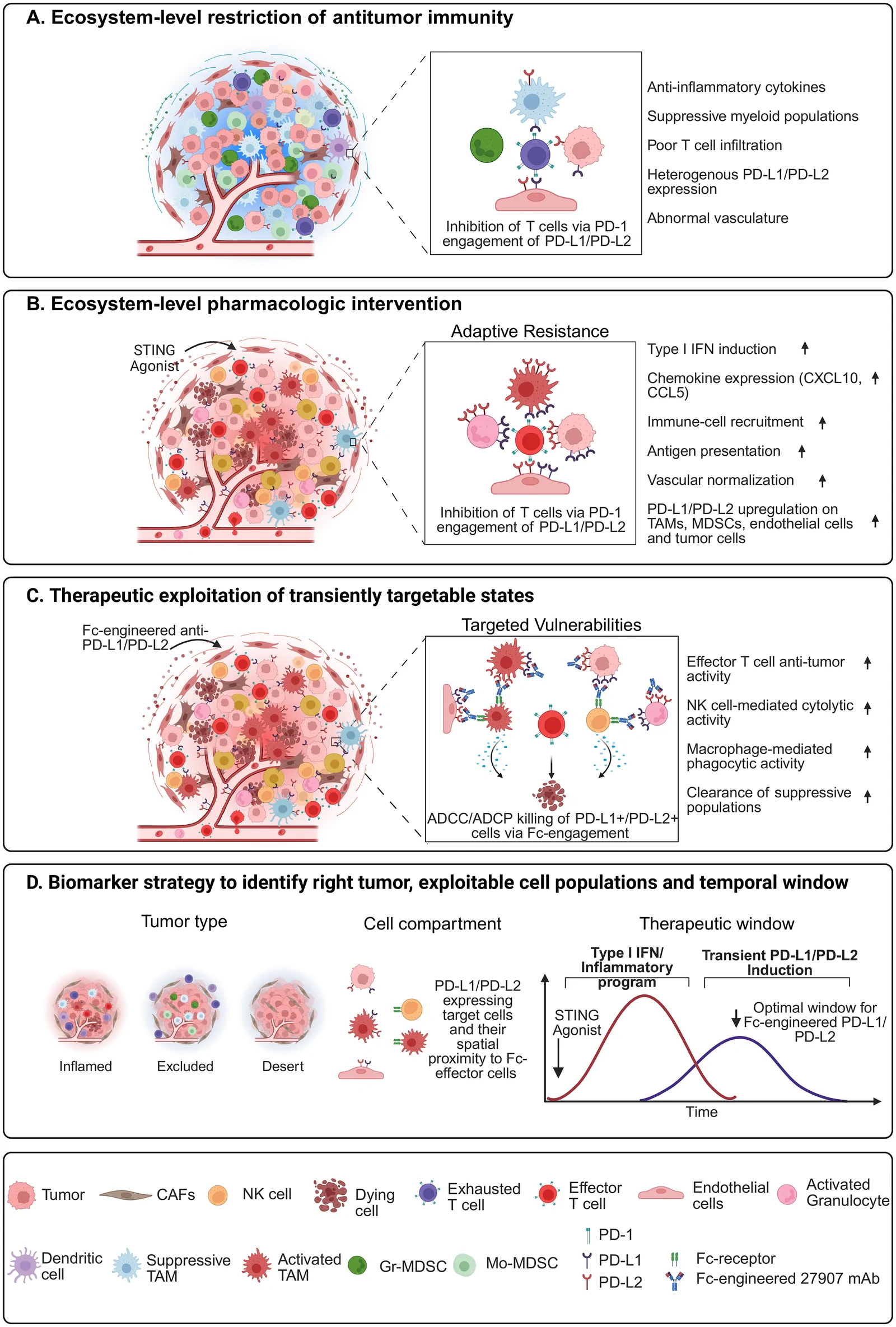
STING-mediated tumor microenvironment remodeling creates both adaptive resistance and targetable vulnerability. **(A)** Baseline resistant tumor ecosystems often exist in an immune-excluded or poorly inflamed state, in which abnormal vasculature, suppressive myeloid populations, stromal barriers, including CAF-driven matrix remodeling, and limited CD8^+^ T-cell access restrict the effectiveness of conventional checkpoint blockade. **(B)** STING agonism can rewire this baseline ecosystem by inducing type I interferon signaling, inflammatory chemokines, immune-cell recruitment, and PD-L1/PD-L2 expression across tumor, myeloid, stromal, and endothelial compartments. **(C)** STING-induced checkpoint-ligand expression creates a biological bifurcation. If left unchecked, PD-L1/PD-L2 upregulation may reinforce adaptive resistance through PD-1-dependent T-cell suppression. However, it may also create a therapeutically targetable state for Fc-engineered anti-PD-L1/PD-L2 antibodies, whereby Fc engagement enables ADCC/ADCP-mediated killing or remodeling of PD-L1/PD-L2-positive cells within suppressive or barrier-forming compartments. **(D)** Biomarker framework illustrating baseline tumor immune states, PD-L1/PD-L2 expression across cell compartments, proximity to FcγR-positive effector cells, and the timing of type I interferon/interferon-stimulated gene induction relative to the transient PD-L1/PD-L2 induction window for Fc-engineered antibody treatment. Translating this strategy requires biomarkers that define the appropriate tumor context, identify the compartment in which PD-L1/PD-L2 is induced, and determine the timing of the STING-induced window of vulnerability. Spatial and pharmacodynamic profiling may therefore help distinguish tumors in which STING priming has primarily induced adaptive resistance from those in which it has created an exploitable therapeutic state. Created with BioRender.com.

Immunotherapy resistance should therefore be considered not only as a failure of T-cell activation, but also as a failure of tissue-level immune organization. Static biomarkers such as tumor mutational burden and baseline PD-L1 expression capture only a partial snapshot of this ecosystem and do not adequately incorporate dynamic and spatial features of the TME (12). The cGAS-STING pathway offers a particularly attractive opportunity in this regard, not because it simply amplifies antitumor immunity, but because it can reshape multiple cellular and spatial features of the TME, including the vascular interface that controls immune-cell entry (**Figure 1B**). By inducing inflammatory remodeling, STING activation may help convert resistant tumor ecosystems into transiently targetable states that need to be understood and therapeutically exploited.

### 1.2 STING agonism as tumor microenvironment reconfiguration

Cytosolic DNA, whether derived from micronuclei (13), mitochondrial leakage (14), or exogenous pathogen-associated sources (15), activates cGAS and promotes the production of the cyclic dinucleotide 2’3’-cGAMP (cyclic [G(2’,5’)pA(3’,5’)p]). 2’3’-cGAMP then binds STING and initiates downstream signaling through TBK1, IRF3, and NF-κB (16–18). The result is a coordinated innate immune response characterized by type I interferon production, secretion of inflammatory chemokines such as CXCL9/10 and CCL5, and activation of transcriptional programs that promote dendritic-cell maturation and CD8^+^ T-cell cross-priming (19–21). This biology is well established and provides the rationale for therapeutic STING activation in cancer, particularly in poorly inflamed tumors in which antigen presentation, chemokine production, and effector T-cell recruitment are insufficient.

What is less often emphasized is the extent to which STING agonism can influence the composition and function of both immune and non-immune compartments, thereby altering the immunological architecture of the TME. Beyond dendritic cells, STING activation can reshape suppressive myeloid populations, including TAMs and MDSCs, toward more inflammatory and less immunosuppressive states (22–25). These changes may increase the production of pro-inflammatory cytokines and chemokines, enhance antigen presentation, and improve the capacity of the TME to support effector immune-cell activity (**Figure 1B**). However, these effects should be interpreted as context-dependent myeloid reprogramming rather than as a uniform or irreversible shift toward a single pro-inflammatory phenotype.

STING-dependent remodeling may also involve non-immune stromal compartments. CAFs can shape the inflammatory state of the TME through extracellular matrix remodeling, cytokine production, and reciprocal interactions with malignant and immune cells. However, their relationship with cGAS-STING signaling appears to be highly context-dependent (26, 27). In colorectal cancer, CAF-rich, stromal VCAN-high tumors have been associated with reduced tumor-cell-intrinsic cGAS and STING expression, and primary CAFs were shown to downregulate cGAS and/or STING in CRC cell lines *in vitro* (28). These findings suggest that tumor crosstalk with CAFs may contribute to a STING-low, immunologically restrained tumor state and provide a rationale for pharmacologic STING activation as a way to bypass or counteract stromal suppression of endogenous cGAS-STING signaling. In selected contexts, CAFs can directly contribute to checkpoint-mediated immune evasion; notably, CAF-mediated antigen cross-presentation has been shown to promote antigen-specific deletion of CD8^+^ T cells through PD-L2 and FASL (29). Thus, although the relative importance of CAFs as direct PD-L1/PD-L2-positive targets is likely to be tumor- and state-dependent, CAFs may contribute both to the regulation of STING responsiveness and to the checkpoint-ligand landscape that shapes the therapeutic window for PD-L1/PD-L2-directed Fc-mediated intervention.

By contrast, tumor-associated endothelial cells may represent a more direct stromal target compartment for STING-induced checkpoint-ligand vulnerability. STING activation in endothelial cells has been shown in preclinical models to drive type I interferon production, support spontaneous antitumor CD8^+^ T-cell responses, and sensitize tumors to ICB (30). Additional studies suggest that endothelial STING signaling can promote features of vascular normalization, including improved perfusion, increased pericyte coverage, and enhanced endothelial-leukocyte interactions, thereby facilitating T-cell trafficking across the vascular barrier (31, 32) These observations position the endothelium not only as a regulator of immune-cell entry, but also as a potential STING-responsive compartment whose inflammatory reprogramming could create therapeutically actionable PD-L1/PD-L2 expression.

Taken as a whole, this evidence supports a broader interpretation of STING agonism. Its therapeutic value should not be reduced to generalized innate immune stimulation or T-cell priming. Rather, STING agonists can be viewed as pharmacological tools capable of transiently reconfiguring the TME, by altering the distribution, phenotype, and vulnerability of immune, stromal, vascular, and malignant compartments. This same biology, however, creates a translational paradox. The inflammatory programs induced by STING may promote dendritic-cell activation, chemokine production, immune-cell recruitment, and interferon signaling, but they may also induce counter-regulatory mechanisms that limit antitumor immunity. In resistant tumors, STING agonism may therefore be best understood as a precision priming strategy: a means of transiently reconfiguring the tumor ecosystem in ways that can generate both adaptive resistance and therapeutically actionable vulnerabilities.

### 1.3 The STING paradox: when adaptive resistance becomes targetable vulnerability

One of the most therapeutically relevant feedback mechanisms triggered by STING activation is the induction of PD-1 ligands, including both PD-L1 and PD-L2, across multiple compartments of the TME. STING-driven type I interferon signaling can directly induce checkpoint-ligand expression, while immune-cell recruitment, T-cell activation, and subsequent IFN-γ production can further amplify this response. Although PD-L1 often shows higher baseline expression and remains the most clinically established biomarker within the PD-1 axis, PD-L2 may also be highly IFN-α/β-responsive and should not be considered a marginal component of STING-induced adaptive resistance. In this way, the same inflammatory circuitry that promotes antitumor immunity can also generate compensatory signals that restrain T-cell function and limit the effectiveness of the immune response (33–36).

PD-L2 has been less extensively studied than PD-L1, but it may be particularly relevant in selected TME compartments, especially myeloid and endothelial cells (37). In addition, CAFs may contribute to immunotherapy resistance and checkpoint-mediated immune suppression in selected contexts, although the evidence for CAFs as major PD-L1/PD-L2-positive target cells remains more limited and heterogeneous (38). This distinction is important because the biological meaning of checkpoint-ligand induction may vary according to the cellular source. PD-L1 and PD-L2 expression on malignant cells may reflect tumor-cell adaptation to immune pressure, whereas expression on myeloid or endothelial cells may identify suppressive or barrier-forming compartments that participate in tissue-level immune resistance. CAFs should therefore be considered primarily as modulators of the immunosuppressive TME, rather than as primary PD-L1/PD-L2-positive target compartments in the context of the mechanism proposed here.

The standard interpretation is that STING-induced checkpoint-ligand expression represents adaptive immune resistance. In this model, tumor and stromal cells exposed to inflammatory pressure may upregulate PD-L1 and PD-L2, thereby limiting T-cell activity through engagement of PD-1. This provides a strong rationale for combining STING agonists with blockade of the PD-1 axis, including PD-1-, PD-L1-, and potentially PD-L2-directed strategies, to prevent inhibitory signaling. However, if PD-L1 and PD-L2 are induced on resistance-promoting TME compartments, such as suppressive myeloid cells or dysfunctional endothelium, simply blocking the PD-1 axis may not be sufficient. In that setting, therapeutic benefit may require not only interruption of inhibitory signaling, but also Fc-mediated killing or remodeling of the PD-L1/PD-L2-positive cells that sustain tissue-level immune resistance.

A critical issue, however, is that PD-L1 and PD-L2 expression should not be interpreted solely as an indication for cell elimination. STING-induced checkpoint-ligand expression may occur on functionally distinct cell populations, including activated antigen-presenting cells, inflamed endothelial cells, suppressive myeloid cells, stromal cells, and malignant cells. Therefore, the therapeutic objective should not be a nonspecific elimination of all PD-L1/PD-L2-positive cells, but the selective killing or remodeling of PD-L1/PD-L2-positive compartments that also display resistance-promoting features (39, 40). In suppressive myeloid populations, for example, checkpoint-ligand induction may coexist with additional immunosuppressive programs, including arginine metabolism, inhibitory cytokine production, reactive oxygen or nitrogen species, and impaired antigen-presenting function (41). Similarly, checkpoint-ligand expression on dysfunctional tumor endothelium may identify vascular barriers that limit T-cell entry or sustain immune exclusion (6). Thus, PD-L1 and PD-L2 should be viewed not as standalone markers of harmful cells, but as components of broader cellular states that need to be resolved by cell-type-specific, spatial, and functional biomarkers.

In the alternative view proposed here, checkpoint-ligand induction is not only a mechanism of adaptive resistance, but also a molecular trace of STING-driven TME reconfiguration. PD-L1 and PD-L2 upregulation may phenotypically mark cells that have responded to STING-induced inflammation and entered a transient, treatment-induced state that may be targetable only when coupled to resistance-promoting cellular programs and their spatial context within the TME (**Figures 1C, D**). The central translational question is therefore not only how to block STING-induced adaptive resistance, but how to identify, leverage, and selectively exploit the harmful compartments within this induced state.

### 1.4 Endothelial and myeloid compartments as targetable mediators of resistance

Tumor endothelial cells and suppressive myeloid cells are central regulators of immune resistance, while CAFs can further reinforce stromal and biochemical barriers to immune infiltration. Tumor endothelial cells are not passive structural scaffolds that simply mediate blood and lymphatic transport. Rather, they actively regulate leukocyte extravasation, shape chemokine gradients within the tumor bed, modulate vascular permeability, and express immunoregulatory surface molecules, including PD-L1 and, in selected contexts, PD-L2, that can influence T-cell entry and cytolytic activity (42, 43). In many solid tumors, dysfunctional endothelium driven by aberrant angiogenesis is a central mechanism of immune exclusion. VEGF-driven angiogenesis promotes structurally abnormal and functionally impaired tumor vessels, leading to hypoperfusion, hypoxia, elevated interstitial pressure, and reduced endothelial permissiveness to leukocyte adhesion and transmigration. As a result, even when T cells are primed in draining lymph nodes, they may fail to efficiently extravasate into the tumor parenchyma if the vascular surface does not support the adhesion and migration programs required for tissue entry, including the expression of molecules such as VCAM-1 and ICAM-1 (44).

Suppressive myeloid cells exacerbate this problem from within the TME. TAMs, MDSCs, and regulatory dendritic-cell states can collectively inhibit T-cell activation, restrict antigen presentation, promote metabolic competition, and elicit inhibitory ligand expression, including PD-L1 and PD-L2 (45). Immunosuppressive and tissue-remodeling TAM states, together with MDSCs, are particularly important in this regard, as they support immune suppression through cytokine production, nutrient depletion, arginine metabolism, reactive oxygen and nitrogen species, and checkpoint-ligand expression (46). These populations may further increase PD-L1 and/or PD-L2 expression after exposure to inflammatory cytokines. CAFs can also contribute to immune exclusion by remodeling the extracellular matrix, shaping cytokine and chemokine gradients, and creating physical and biochemical barriers to T-cell infiltration; however, current evidence for CAFs as dominant PD-L1/PD-L2-positive target cells is more limited and context-dependent than for myeloid or endothelial compartments. In this setting, checkpoint-ligand expression is not restricted to malignant cells but is distributed across multiple non-malignant compartments that contribute to immune suppression.

This distinction is essential because the biological meaning of PD-L1 and PD-L2 expression depends on the cell type in which they are expressed. Checkpoint-ligand induction on tumor cells may primarily reflect malignant-cell adaptation to immune pressure, whereas induction on myeloid or endothelial cells may mark suppressive or barrier-forming elements of the TME. In the latter case, PD-L1 and PD-L2 may function not only as inhibitory ligands but also as surface markers of resistant, non-malignant compartments that could be selectively targeted after inflammatory priming. CAFs should therefore be considered important regulators of the stromal and inflammatory context in which this process occurs, rather than primary target compartments in the mechanism proposed here.

The endothelial and myeloid compartments may therefore represent actionable mediators of resistance. STING activation can remodel these compartments and may expose them to immune-mediated targeting by inducing or increasing PD-L1 and PD-L2 expression. This concept has important therapeutic implications. If STING-induced PD-L1/PD-L2 expression marks resistance-promoting endothelial or myeloid compartments, then antibody format becomes central: blocking-only antibodies and Fc-enabled antibodies may have fundamentally different consequences for the TME.

Our recently published work (36) provides a mechanistic example of this concept, showing that STING agonism promotes TME remodeling and sensitizes endothelial cells to Fc-engineered anti-PD-L1/PD-L2 targeting. More broadly, these findings support the idea that STING-induced TME reconfiguration can transform resistant stromal and immune-suppressive compartments into therapeutically actionable targets.

### 1.5 Fc-engineered PD-L1/PD-L2 antibodies: converting checkpoint ligands into therapeutic targets

If STING-induced PD-L1 and PD-L2 expression is interpreted as a transient, targetable state, then antibody design becomes critical. The Fc domain of an antibody determines how it engages Fc receptors on immune effector cells and therefore influences whether antigen-positive cells are merely functionally blocked or actively eliminated. Among human IgG subclasses, IgG1 has strong intrinsic capacity to mediate antibody-dependent cellular cytotoxicity (ADCC) and antibody-dependent cellular phagocytosis (ADCP) through engagement of Fcγ receptor-expressing cells, including NK cells, macrophages, monocytes, and other myeloid effectors. However, even within the IgG1 subclass, the magnitude and quality of these effector functions depend on Fc glycosylation, Fcγ receptor affinity, Fcγ receptor polymorphisms, antigen density, and the composition of the local immune microenvironment.

This distinction is particularly important for immune checkpoint antibodies. Many approved checkpoint inhibitors were designed primarily to block inhibitory receptor–ligand interactions rather than to deplete checkpoint-expressing cells. In particular, several PD-1-directed antibodies use IgG4 backbones or Fc modifications that limit Fc-mediated depletion of activated T cells. Similarly, some PD-L1-directed antibodies retain limited or engineered Fc activity depending on their intended mechanism of action. Thus, the Fc domain can profoundly influence the biological activity of checkpoint antibodies and determine whether checkpoint-ligand-positive cells are only functionally inhibited or also targeted for immune-mediated elimination.

Fc engineering can enhance this effector potential. Mutations such as GASDIE, which increase binding to Fcγ receptors including FcγRIIIa and FcγRIIa, can markedly augment ADCC and ADCP relative to parental IgG1 antibodies (47). This gives Fc-engineered anti-PD-L1/PD-L2 antibodies a qualitatively different function from Fc-silent or weakly Fc-active antibodies. Rather than merely blocking PD-1 signaling, they may recruit effector cells to eliminate or remodel PD-L1/PD-L2-positive suppressive compartments within the TME.

In a STING-primed TME, this distinction may be especially relevant. Interferon-driven checkpoint-ligand induction can increase PD-L1 and PD-L2 expression on myeloid, stromal, and endothelial cells, potentially increasing the target density required for efficient Fc-mediated effector mechanisms (**Figure 1C**). Thus, Fc engineering provides the mechanistic bridge between STING-induced adaptive resistance and therapeutic vulnerability. By enabling effector-cell engagement against PD-L1/PD-L2-positive compartments, Fc-competent antibodies may convert a compensatory immune-suppressive response into an opportunity for active TME remodeling.

Although this Perspective focuses on PD-L1 and PD-L2 (36), the same Fc-engineering principle may extend to other immunoregulatory surface molecules expressed in STING-responsive tumor compartments. B7-H3/CD276 is a relevant example because it is enriched on malignant cells and tumor vasculature, and Fc-optimized anti-B7-H3 antibodies can mediate cytotoxic activity (48, 49). Recent preclinical work also reported increased B7-H3 protein expression in MDSCs and bone marrow-derived macrophages following STING agonism, suggesting that B7-H3 may become an inducible target in selected myeloid contexts (50). However, this evidence remains context-specific and does not establish interchangeability with PD-L1/PD-L2. Extending the proposed strategy to B7-H3 or other targets will require demonstration of STING-dependent target induction, sufficient antigen density on resistance-promoting compartments, and an acceptable normal-tissue expression profile.

### 1.6 Biomarkers to define the STING-induced window of vulnerability

A precision approach based on STING priming and checkpoint-ligand targeting requires biomarkers that address two related but distinct questions. The first is a baseline selection question: which tumors are most likely to benefit from STING-mediated priming? The second is a pharmacodynamic question: once a STING agonist has been administered, has the tumor entered the transient state in which PD-L1/PD-L2-positive compartments can be effectively targeted by an Fc-engineered antibody? The distinction is essential because the proposed strategy depends not only on tumor classification at baseline, but also on the induction of a treatment-dependent window of vulnerability. At baseline, STING priming may be most rational in tumors that are poorly responsive to conventional ICB because they are immune-cold, immune-excluded, myeloid-rich, and/or vascularly dysfunctional, but still retain the capacity to mount an interferon-associated inflammatory response. Candidate baseline features may include evidence of intact or inducible cGAS-STING pathway competence, low-to-intermediate pre-existing T-cell infiltration, immune exclusion rather than complete absence of immune priming, suppressive myeloid enrichment, abnormal or non-permissive tumor vasculature, and limited but inducible PD-L1/PD-L2 expression. Tumors that are already highly inflamed and responsive to ICB may not require STING priming, whereas tumors with profound defects in antigenicity, dendritic-cell function, or interferon responsiveness may fail to convert even after STING activation. Therefore, baseline stratification should aim to identify tumors that are resistant but still reprogrammable (51, 52).

This baseline selection problem also needs to be considered in light of the clinical limitations of STING agonists. Despite strong preclinical rationale, STING agonists have shown limited clinical activity as monotherapies or empiric combinations to date, in part because of poor pharmacokinetics, limited systemic activity, challenges in achieving tumor-localized pathway activation, and the risk of systemic inflammatory toxicity. Many first-generation STING agonists have therefore been administered intratumorally, which restricts their use to accessible lesions and may not adequately address disseminated or poorly accessible disease. These limitations argue against using STING agonists as nonspecific immune stimulants and instead support their development as biomarker-guided priming agents, particularly in tumors where local reprogramming of the TME could expose targetable PD-L1/PD-L2-positive compartments (53–55).

Baseline classification alone, however, is unlikely to be sufficient. The success of a cytotoxic Fc-engineered antibody may depend on its administration within the STING-induced window of target expression (**Figure 1D**). Defining when this window opens, how long it persists, and which cell types it affects is therefore a central translational priority. Biomarkers should not simply predict baseline sensitivity; they should also confirm that STING activation has occurred, identify which compartments have been reconfigured, and determine whether PD-L1 and PD-L2 expression has emerged on cells that can be targeted by an Fc-engineered antibody (52, 56).

On-treatment biomarkers are likely to be particularly informative. These may include induction of interferon-stimulated genes, circulating chemokines such as CXCL10 as indicators of interferon pathway activation, PD-L1 and PD-L2 upregulation in accessible tumor tissue or circulating myeloid populations, and evidence of myeloid rewiring in tumor biopsies or draining lymph nodes (53). Endothelial activation should also be assessed when vascular targeting is expected. Potential markers may include increased expression of leukocyte adhesion molecules such as ICAM-1, VCAM-1, or E-selectin, changes in angiogenic or vascular-normalization markers, altered perfusion, and circulating soluble adhesion molecules or angiogenic factors (6, 57). Such biomarkers could help define whether STING activation has converted a resistant tumor into a more inflamed, vascularly permissive, and targetable state.

Spatial resolution matters as much as molecular quantification, but it should be interpreted within the temporal and functional context of STING-induced remodeling. PD-L1 and PD-L2 induction may rise and fall after treatment, and the biological significance of this induction depends on where it occurs. Checkpoint-ligand expression on tumor endothelium occupies a different anatomical and functional niche than expression on suppressive myeloid populations or malignant cells. For Fc-mediated targeting to occur, Fc receptor-positive effector cells must also be present and positioned near the relevant target compartment, where they can mediate ADCC or ADCP (56, 58).

The STING-induced window of vulnerability is therefore best understood not as a single time point, but as a convergence of temporal, spatial, cellular, and functional states. Biomarker strategies that address all these dimensions will be needed to identify the tumors most suitable for STING priming, confirm that pharmacodynamic reconfiguration has occurred, and determine the optimal moment at which Fc-engineered PD-L1/PD-L2 targeting is most likely to be effective.

Clinical translation will also be complicated by intralesional, interlesional, cellular, and temporal heterogeneity. A single core biopsy may not capture regional differences in PD-L1/PD-L2 induction within a lesion, while local STING administration may produce different pharmacodynamic effects in injected and distant tumors (53, 59). Bulk checkpoint-ligand scoring may also obscure whether expression occurs on malignant, endothelial, myeloid, or antigen-presenting cells, and these treatment-induced states may evolve over different timescales (39, 40, 60). Biomarker sampling must therefore be spatially and temporally aligned with the proposed therapeutic window.

A clinically tractable early-phase strategy could combine a baseline biopsy with one predefined early on-treatment biopsy from an accessible lesion, paired blood sampling, and, where feasible, comparison of injected and uninjected lesions. A focused assay panel could include compartment-resolved PD-L1/PD-L2 expression by multiplex immunohistochemistry or immunofluorescence, an interferon-stimulated gene or CXCL10 pharmacodynamic readout, markers of endothelial and myeloid reprogramming, and the proximity of Fcγ receptor-positive effector cells. Rather than relying on a single biomarker, a composite go/no-go criterion could confirm STING pathway activation, target induction in resistance-promoting compartments, and effector-cell accessibility before Fc-engineered antibody treatment.

### 1.7 STING delivery platforms: translational considerations

The translational development of STING-mediated TME reconfiguration is partly constrained by drug-delivery considerations. Intratumoral administration can enable local STING activation while reducing systemic exposure, but it limits treatment to accessible lesions and may not adequately address disseminated or poorly accessible disease. Conversely, systemic administration remains challenging for many STING agonists. In particular, first-generation cyclic dinucleotide agonists may be limited by rapid clearance, poor membrane permeability, inefficient cytosolic delivery, and insufficient tumor accumulation, whereas broader systemic pathway activation may increase the risk of inflammatory toxicity. These limitations are particularly relevant to the strategy proposed here, because its therapeutic effect is expected to depend on controlled, tumor-localized, and time-limited STING activation rather than generalized innate immune stimulation.

Nano-immuno-oncology encompasses several complementary approaches that may inform the development of more spatially and temporally controlled immunotherapies. In an orthotopic glioblastoma model, glycosylated dendrimer nanoamplifiers designed to exploit DNA damage–immune crosstalk illustrate how nanocarrier engineering may facilitate blood–brain barrier penetration, tumor accumulation, and coordinated radio-immunotherapy (61).

A variety of nanocarrier platforms have been developed across therapeutic modalities to overcome delivery barriers (62, 63). In the specific context of STING agonism, distinct nanocarrier designs have been investigated to address the unique challenges of intracellular delivery and tumor targeting (64–66).

Targeted nanocarriers provide a particularly relevant proof of concept. In an orthotopic glioblastoma model, a CD47/PD-L1-targeting lipid nanoparticle carrying the STING agonist diABZI was designed to engage tumor-associated myeloid cells and glioma cells, enhance myeloid-cell phagocytic activity, activate STING signaling in tumor-associated myeloid cells, and increase subsequent T-cell recruitment (67). Although this platform differs from the sequential STING-priming and Fc-engineered antibody strategy proposed here, it demonstrates that checkpoint-associated surface molecules can be exploited to direct STING agonist delivery toward defined TME compartments.

Nanocarrier-based STING delivery could be compatible with the treatment-induced window of vulnerability proposed in this Perspective. Beyond their potential to induce a targetable state, these formulations should be evaluated for their ability to activate STING at the appropriate location and time to support effective combination therapy. Such activation could, in principle, localize type I interferon signaling, chemokine induction, and endothelial reprogramming within selected tumor compartments (30). Our recent study further showed that local STING agonism can induce PD-L1/PD-L2 expression in TME compartments, thereby creating a potentially targetable state for subsequent treatment with an Fc-engineered antibody (36). Whether nanocarriers can reproducibly generate and synchronize this state will depend on their biodistribution, cellular tropism, intracellular trafficking, payload-release kinetics, and the baseline biological features of the treated tumor (68).

Related targeted-delivery modalities may also inform this strategy. XMT-2056, a HER2-directed STING agonist antibody–drug conjugate, achieved tumor-targeted agonist delivery following systemic administration, activated cancer cells and tumor-resident immune cells, and demonstrated antitumor activity across models with different levels of HER2 expression, while reducing systemic inflammatory effects compared with a free STING agonist (69). Complementary mechanistic studies using tumor cell-directed STING agonist antibody-drug conjugates showed that tumor-antigen engagement can facilitate FcγRI-mediated uptake of the conjugate by myeloid cells, thereby extending STING payload delivery beyond antigen-positive tumor cells and contributing to innate antitumor responses (70), Although antibody–drug conjugates are distinct from conventional nanocarrier platforms, these studies illustrate how antigen targeting and Fc biology can jointly influence the spatial and cellular distribution of a STING payload. They also raise questions regarding baseline target abundance, internalization, intracellular payload release, and coordination between STING activation and subsequent checkpoint-ligand targeting (70).

STING delivery platforms would not eliminate the need for biomarker guidance. It would remain necessary to establish whether STING activation has occurred at the appropriate site and intensity, which cellular compartments have responded, and whether a therapeutically relevant PD-L1/PD-L2-positive state has been generated. Studies of novel STING delivery platforms should therefore incorporate pharmacodynamic and spatial measurements, including interferon-stimulated gene induction, chemokine production, endothelial activation or vascular remodeling, myeloid reprogramming, and compartment-specific PD-L1/PD-L2 expression. Biodistribution, activation kinetics, pharmacokinetics, and cellular targeting should also be considered integral components of the biomarker strategy rather than merely formulation characteristics (68). These platforms should therefore be viewed as potentially complementary means of improving the spatial and temporal control of STING priming. Their ability to generate and support the proposed therapeutic window will require dedicated preclinical and clinical validation.

## 2 Discussion

The same context-dependent biology that makes STING-mediated TME reconfiguration attractive also creates translational uncertainty. If STING activation can induce a targetable inflammatory state within tumors, similar inflammatory or checkpoint-ligand changes may also occur in normal tissues, especially under conditions of systemic exposure, pre-existing inflammation, or insufficient tumor localization (52). This concern may be reduced, but not eliminated, by local intratumoral STING administration. Indeed, our recently published work provides a model in which local STING agonism is used to reconfigure the TME, while systemic administration of an Fc-engineered anti-PD-L1/PD-L2 antibody exploits the induced target state (36). Targeted STING delivery formulations may offer an additional means of biasing pathway activation toward tumor tissue or selected TME compartments, although their capacity to achieve effective tumor localization while avoiding off-target pathway activation remains to be established (65, 66). In this setting, the objective is not maximal STING activation, but the controlled induction of a tumor-localized and time-limited vulnerability that can be therapeutically exploited before it becomes harmful, self-limiting, or systemically inflammatory.

A central challenge will therefore be to define the tumor types, delivery modalities, timing, and treatment sequences for which this strategy is most likely to be effective. STING activation may create a transient window during which PD-L1 and PD-L2 are induced on targetable TME compartments, but the magnitude and relevance of this window will vary across tumor contexts. Tumors characterized by immune exclusion, suppressive myeloid enrichment, abnormal vasculature, and limited response to conventional ICB may be particularly suitable candidates, provided that they retain sufficient STING responsiveness and can be effectively primed locally or through tumor-targeted delivery (6, 51). Conversely, highly inflamed tumors already responsive to ICB may not require STING priming, whereas deeply immune-desert tumors with defective antigenicity, impaired dendritic-cell function, or poor interferon responsiveness may fail to convert into a targetable state.

The delivery platform will itself influence the onset, intensity, and duration of STING-mediated reconfiguration. An intratumoral bolus, a locally retained formulation, and a systemically administered targeted-delivery system may generate substantially different pharmacodynamic profiles (64–66). Because the magnitude and scheduling of STING activation can influence the balance between local tumor control and durable adaptive immunity, controlled release should not be assumed to be intrinsically advantageous. Prolonged or poorly synchronized pathway activation could blur the desired transient window or complicate the timing of antibody administration (71, 72). The dose, formulation, release kinetics, and sequence of STING agonists and Fc-engineered antibodies should therefore be optimized according to tumor type and pharmacodynamic evidence rather than empirical combination alone.

These delivery and timing considerations are closely linked to safety. The combination strategy introduces two mechanistically distinct sets of risks. First, although STING activation is required for therapeutic efficacy, inflammatory signaling should remain spatially restricted, because systemic pathway activation can cause cytokine-mediated toxicity, whose severity depends on the agent, route, dose, and extent of systemic exposure. Local STING administration is expected to reduce systemic cytokine release and off-tumor inflammatory activation relative to untargeted systemic exposure. Intratumoral MIW815/ADU-S100 was generally well tolerated, with no maximum tolerated dose reached; pyrexia, chills, and injection-site pain were among the most common treatment-related adverse events (53). However, rapid absorption into plasma and induction of circulating cytokines indicated that intratumoral delivery reduces but does not fully confine pathway activation. More potent intratumoral STING agonists have produced dose-limiting toxicities (73), underscoring that systemic symptoms and cytokine-mediated toxicity remain important considerations at higher exposures or with more potent agents. When STING agonists are combined with checkpoint-directed antibodies, conventional organ-specific immune-related adverse events associated with PD-1-axis blockade should also be monitored. Tumor-biased delivery formulations may provide additional spatial control, but neither local nor targeted delivery guarantees tumor confinement. Nanocarrier biodistribution, off-target uptake, and activation of circulating or tissue-resident myeloid populations should therefore be evaluated alongside conventional pharmacodynamic measurements (65, 66). Tumor vessels differ from normal vasculature in their structure, activation state, permeability, and exposure to angiogenic and inflammatory cues; these differences may create a therapeutic window for targeting tumor-associated endothelium (6). Nevertheless, endothelial cells in normal tissues can also respond to inflammatory stimuli, and PD-L1/PD-L2 induction outside the tumor cannot be excluded, particularly in inflamed or damaged tissues.

Fc-mediated depletion introduces a second, mechanistically distinct safety concern. The most relevant clinical evidence is partially reassuring: avelumab, an unengineered IgG1 anti-PD-L1 antibody that retains ADCC activity, produced no significant changes across 123 peripheral immune-cell subsets over multiple treatment cycles, including PD-L1-positive populations (74). These findings support the feasibility of preserving Fc effector function when targeting the PD-L1 axis, but they do not establish the safety of GASDIE-enhanced antibodies targeting STING-primed endothelial and myeloid compartments. Risk mitigation should therefore be incorporated into preclinical and early clinical development by comparing Fc-silent, parental IgG1, and Fc-enhanced formats; defining target-density and depletion thresholds across malignant, suppressive, activated immune, and normal endothelial cells; using sequential or step-up dosing; and monitoring circulating immune subsets, endothelial injury, coagulation, and organ-specific inflammation. A mechanism-guided sequence in which local or tumor-restricted STING priming is followed by systemic antibody treatment after the peak of STING-induced cytokine signaling, as modeled in our recent study, provides a testable starting strategy, but not proof of safety (36). The aim is to define the boundary between productive tumor-localized remodeling and unacceptable vascular, inflammatory, or immune-mediated toxicity.

Future studies should be designed to test the central hypothesis that STING-induced adaptive resistance can be converted into therapeutic vulnerability. This will require defining the timing, location, and cellular source of PD-L1 and PD-L2 induction following either intratumoral administration or systemic targeted-delivery of STING agonists, including nanoparticle- and antibody conjugate-based approaches, and determining whether these induced compartments can be safely and effectively targeted by Fc-engineered antibodies. Spatial transcriptomics, multiplex immunofluorescence, imaging mass cytometry, and single-cell profiling could be used to map checkpoint-ligand induction by cellular compartment and anatomical location (58). These methods may help determine whether PD-L1 and PD-L2 are induced primarily on malignant cells, myeloid cells, endothelial cells, CAFs, or other stromal populations, and whether these cells are positioned in regions accessible to Fc receptor-positive effector cells. For systemic targeted-delivery approaches, spatial profiling should be integrated with measurements of biodistribution, cellular tropism, intracellular delivery, payload-release kinetics, and pathway activation (65, 67). This would establish not simply whether the formulation reaches the tumor, but whether it induces the required biological state in the appropriate target compartment.

It will also be important to compare Fc-silent and Fc-competent checkpoint-ligand antibodies in defined TME contexts. Fc-mediated effector function should not be assumed to be universally beneficial. Its value will depend on whether PD-L1/PD-L2 expression marks resistance-promoting compartments, such as suppressive myeloid cells or dysfunctional endothelium, rather than beneficial effector or antigen-presenting immune cells or vulnerable normal tissues. Target selection should therefore be informed by cellular identity, spatial localization, and coexisting resistance programs rather than by checkpoint-ligand expression alone. These comparisons should first be addressed in appropriate preclinical models that preserve relevant vascular, myeloid, and stromal features of the TME before being translated into clinical strategies. Such studies could clarify when Fc-enabled activity improves TME remodeling and when it may instead increase toxicity.

Tumors with highly immunosuppressive, vascularized, or myeloid-rich microenvironments may be particularly relevant settings in which to test this concept. Glioblastoma could represent an informative disease context, given its profound immunosuppression, myeloid enrichment, abnormal vasculature, and resistance to conventional ICB (75). Systemic targeted-delivery strategies may be particularly relevant in this setting because they can be engineered to influence brain and tumor delivery, cellular targeting, and local payload release. However, the heterogeneous blood-brain and blood-tumor barriers, the unique central nervous system immune environment, and the risk of inflammation-associated edema would require a dedicated and cautious translational strategy (61, 67).

Beyond conventional formulations, a more speculative extension of this concept would be the development of checkpoint-ligand-targeted STING agonist conjugates (69, 70, 76). In principle, an Fc-engineered dual PD-L1/PD-L2 antibody could serve both as an effector-competent checkpoint-ligand-targeting agent and as a vehicle for localized STING activation within PD-L1/PD-L2-positive TME niches. However, such a design differs fundamentally from the sequential strategy proposed here because the same molecule would be expected both to generate and to exploit the targetable state. If efficient delivery initially requires PD-L1/PD-L2 expression, while the payload itself is intended to induce or amplify these ligands, the approach may involve a circular dependence on baseline target density, internalization, and payload release. Fc-mediated elimination of target-positive cells would also need to be coordinated with intracellular STING activation. These considerations raise unresolved questions regarding treatment timing, feed-forward checkpoint-ligand induction, cellular uptake, and off-tumor inflammatory activation, and justify treating this modality as a separate, hypothesis-generating strategy rather than a direct substitute for sequential STING priming and antibody administration.

In conclusion, STING agonism provides an opportunity to rethink how precision immuno-oncology can overcome resistance. Rather than acting solely as an innate immune stimulant, STING activation can reconfigure the TME, generating both compensatory resistance and therapeutic vulnerability. The induction of PD-L1 and PD-L2 after STING activation, traditionally interpreted as adaptive immune escape, may become pharmacologically exploitable when paired with Fc-engineered antibodies capable of checkpoint blockade and effector-mediated remodeling. Systemic targeted delivery may help extend this framework by providing additional control over the localization, cellular distribution, and kinetics of STING activation, but its value will depend on whether it can reproducibly generate the required targetable state. Future development should therefore focus on defining the tumor types, delivery modalities, dosing schedules, and cellular compartments in which the window of vulnerability emerges, while integrating biodistribution, vascular, inflammatory, immune, and spatial biomarkers into preclinical and early translational studies.
